# Supplementary figures and images for: Risk factor control and cardiovascular events in patients with type 2 diabetes mellitus
Source: PLoS One. 2024 Feb 29;19(2):e0299035. doi: 10.1371/journal.pone.0299035 (PMC10903792; doi:10.1371/journal.pone.0299035)

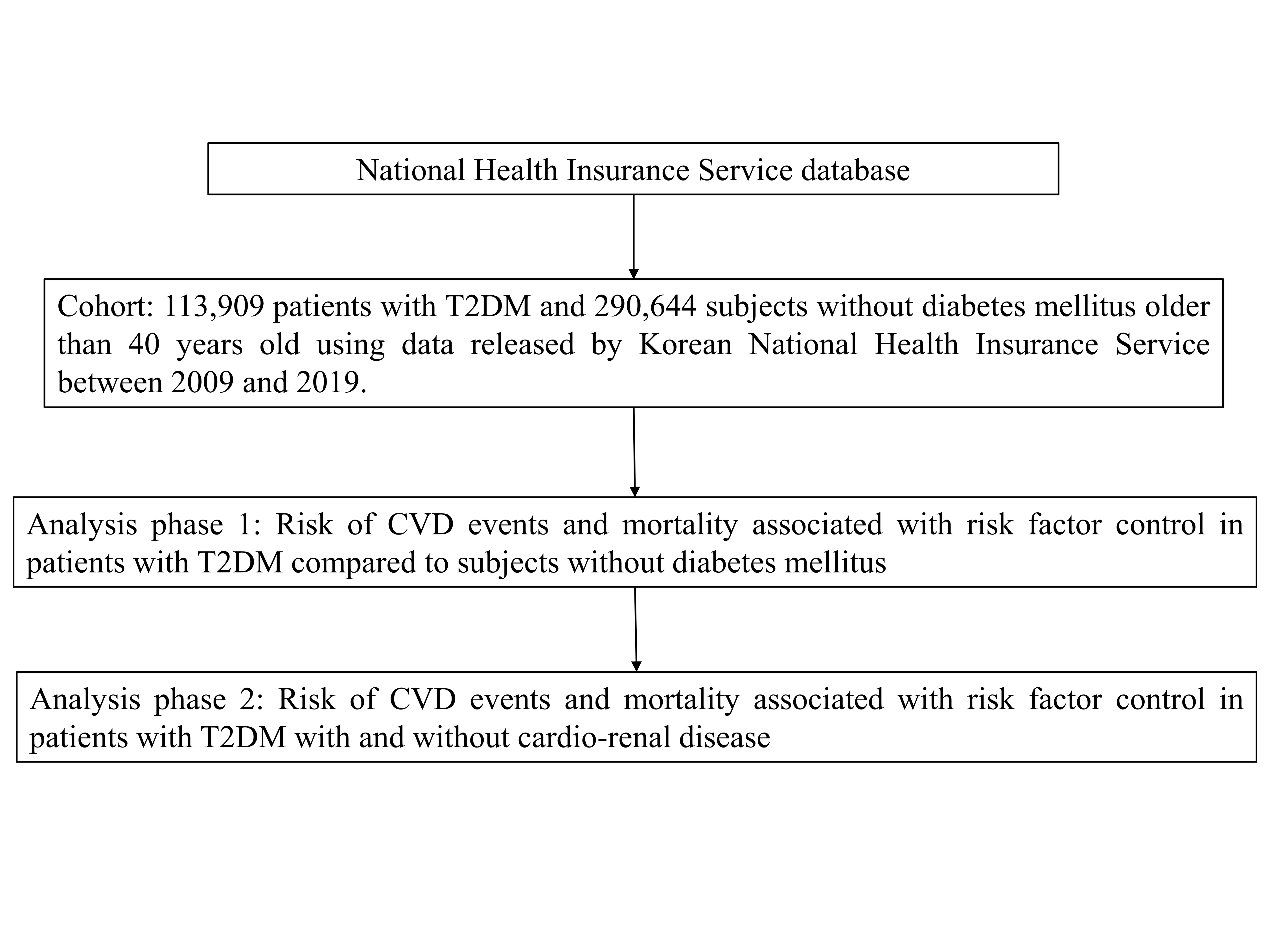

Supplement: S1 Fig — CVD, cardiovascular disease. (TIF) [file pone.0299035.s001.tif]
