# Supplementary material for: Risk factor control and cardiovascular events in patients with type 2 diabetes mellitus
Source: PLoS One. 2024 Feb 29;19(2):e0299035. doi: 10.1371/journal.pone.0299035 (PMC10903792; doi:10.1371/journal.pone.0299035)
Supplement: S1 Table — Data are presented as frequencies and proportions. CVD, cardiovascular disease. (DOCX) [file pone.0299035.s002.docx]

**S1 Table. Cardiovascular outcomes of study participants.**

|  | Diabetic patients | | Subjects without diabetes |
| --- | --- | --- | --- |
| CVD events, n (%) | 14,184 (12.5) | 18,476 (6.4) | |
| Coronary events, n (%) | 7,583 (6.7) | 9,573 (3.3) | |
| Cerebrovascular events, n (%) | 6,298 (5.5) | 8,564 (3.0) | |
| Heart failure hospitalization, n (%) | 1,401 (1.2) | 1,189 (0.4) | |
| CVD mortality, n (%) | 1,766 (1.6) | 1,896 (0.7) | |
| Coronary heart disease mortality, n (%) | 772 (0.7) | 795 (0.3) | |
| Stroke mortality, n (%) | 763 (0.7) | 879 (0.3) | |
| Heart failure mortality, n (%) | 231 (0.2) | 222 (0.1) | |

Data are presented as frequencies and proportions.

CVD, cardiovascular disease.
