# Supplementary material for: Risk factor control and cardiovascular events in patients with type 2 diabetes mellitus
Source: PLoS One. 2024 Feb 29;19(2):e0299035. doi: 10.1371/journal.pone.0299035 (PMC10903792; doi:10.1371/journal.pone.0299035)
Supplement: S2 Table — Hazard ratios were adjusted for age, gender, follow-up, history of cardiovascular disease, and prescriptions for hypoglycemic, antihypertensive, and lipid-lowering therapy. HR, hazard ratio; CI, confidence interval. (DOCX) [file pone.0299035.s003.docx]

**S2 Table. The relative risk of coronary events in participants according to the degree of risk factor.**

|  |  | Uncontrolled risk factors, N | Total  cases | Events | Person-years | Incidence rate per 1000 person-years (95% CI) | HR | 95% CI | P-value |
| --- | --- | --- | --- | --- | --- | --- | --- | --- | --- |
| Total  participants | Subjects without diabetes |  | 290,339 | 9,573 | 2,655,823 | 3.6 (3.5-3.7) |  |  |  |
|  | Patients with diabetes | 0 | 8,280 | 659 | 65,108 | 10.1 (9.3-10.9) | 1.22 | 1.13-1.33 | <0.001 |
|  |  | 1 | 45,253 | 2,500 | 385,955 | 6.5 (6.2-6.7) | 1.08 | 1.03-1.14 | 0.001 |
|  |  | 2 | 38,348 | 2,568 | 324,676 | 7.9 (7.6-8.2) | 1.21 | 1.15-1.27 | <0.001 |
|  |  | 3 | 17,264 | 1,444 | 143,654 | 10.1 (9.5-10.6) | 1.46 | 1.37-1.55 | <0.001 |
|  |  | ≥4 | 4,764 | 412 | 39,357 | 10.5 (9.5-11.5) | 1.48 | 1.33-1.64 | <.001 |
| Patients with diabetes | | 0 | 8,280 | 659 | 65,108 | 10.1 (9.3-10.9) |  |  |  |
|  |  | 1 | 45,253 | 2,500 | 385,955 | 6.5 (6.2-6.7) | 0.87 | 0.80-0.95 | 0.001 |
|  |  | 2 | 38,348 | 2,568 | 324,676 | 7.9 (7.6-8.2) | 0.97 | 0.89-1.06 | 0.515 |
|  |  | 3 | 17,264 | 1,444 | 143,654 | 10.1 (9.5-10.6) | 1.18 | 1.07-1.29 | 0.001 |
|  |  | ≥4 | 4,764 | 412 | 39,357 | 10.5 (9.5-11.5) | 1.20 | 1.06-1.36 | 0.004 |
| Patients with diabetes with cardio-renal disease | | 0 | 4,859 | 569 | 35,748 | 15.9 (14.6-17.2) |  |  |  |
|  |  | 1 | 21,305 | 1,749 | 172,653 | 10.1 (9.7-10.6) | 0.81 | 0.74-0.89 | <0.001 |
|  |  | 2 | 18,147 | 1,708 | 146,630 | 11.6 (11.1-12.2) | 0.87 | 0.79-0.96 | 0.004 |
|  |  | 3 | 7,698 | 886 | 60,540 | 14.6 (13.7-15.6) | 1.01 | 0.91-1.13 | 0.813 |
|  |  | ≥4 | 1,849 | 227 | 14,438 | 15.7 (13.7-17.8) | 1.01 | 0.86-1.18 | 0.919 |
| Patients with diabetes without cardio-renal disease | | 0 | 3,421 | 90 | 29,360 | 3.1 (2.4-3.7) |  |  |  |
|  |  | 1 | 23,948 | 751 | 213,302 | 3.5 (3.3-3.8) | 1.34 | 1.07-1.66 | 0.010 |
|  |  | 2 | 20,201 | 860 | 178,046 | 4.8 (4.5-5.2) | 1.64 | 1.32-2.03 | <0.001 |
|  |  | 3 | 9,566 | 558 | 83,114 | 6.7 (6.2-7.3) | 2.09 | 1.67-2.61 | <0.001 |
|  |  | ≥4 | 2,915 | 185 | 24,918 | 7.4 (6.4-8.5) | 2.16 | 1.68-2.79 | <0.001 |

Hazard ratios were adjusted for age, gender, follow-up, history of cardiovascular disease, and prescriptions for hypoglycemic, antihypertensive, and lipid-lowering therapy.

HR, hazard ratio; CI, confidence interval.
