# Supplementary material for: Risk factor control and cardiovascular events in patients with type 2 diabetes mellitus
Source: PLoS One. 2024 Feb 29;19(2):e0299035. doi: 10.1371/journal.pone.0299035 (PMC10903792; doi:10.1371/journal.pone.0299035)
Supplement: S3 Table — Hazard ratios were adjusted for age, gender, follow-up, history of cardiovascular disease, and prescriptions for hypoglycemic, antihypertensive, and lipid-lowering therapy. HR, hazard ratio; CI, confidence interval. (DOCX) [file pone.0299035.s004.docx]

**S3 Table. The relative risk of cerebrovascular events in participants according to the degree of risk factor.**

|  |  | Uncontrolled risk factors, N | Total  cases | Events | Person-years | Incidence rate per 1000 person-years (95% CI) | HR | 95% CI | P-value |
| --- | --- | --- | --- | --- | --- | --- | --- | --- | --- |
| Total  participants | Subjects without diabetes |  | 290,339 | 8,564 | 2,667,130 | 3.2 (3.1-3.3) |  |  |  |
|  | Patients with diabetes | 0 | 8,280 | 437 | 66,917 | 6.5 (5.9-7.1) | 0.94 | 0.85-1.03 | 0.188 |
|  |  | 1 | 45,253 | 2,114 | 390,161 | 5.4 (5.2-5.6) | 0.98 | 0.93-1.03 | 0.330 |
|  |  | 2 | 38,348 | 2,217 | 328,231 | 6.8 (6.5-7.0) | 1.15 | 1.09-1.21 | <0.001 |
|  |  | 3 | 17,264 | 1,155 | 145,976 | 7.9 (7.5-8.4) | 1.39 | 1.30-1.49 | <0.001 |
|  |  | ≥4 | 4,764 | 375 | 39,722 | 9.4 (8.5-10.4) | 1.87 | 1.68-2.09 | <0.001 |
| Patients with diabetes | | 0 | 8,280 | 437 | 66,917 | 6.5 (5.9-7.1) |  |  |  |
|  |  | 1 | 45,253 | 2,114 | 390,161 | 5.4 (5.2-5.6) | 1.03 | 0.93-1.14 | 0.603 |
|  |  | 2 | 38,348 | 2,217 | 328,231 | 6.8 (6.5-7.0) | 1.22 | 1.10-1.35 | <0.001 |
|  |  | 3 | 17,264 | 1,155 | 145,976 | 7.9 (7.5-8.4) | 1.48 | 1.32-1.65 | <0.001 |
|  |  | ≥4 | 4,764 | 375 | 39,722 | 9.4 (8.5-10.4) | 1.98 | 1.72-2.28 | <0.001 |
| Patients with diabetes with cardio-renal disease | | 0 | 4,859 | 337 | 37,505 | 9.0 (8.0-9.9) |  |  |  |
|  |  | 1 | 21,305 | 1,389 | 176,279 | 7.9 (7.5-8.3) | 1.01 | 0.90-1.14 | 0.840 |
|  |  | 2 | 18,147 | 1,381 | 149,471 | 9.2 (8.8-9.7) | 1.14 | 1.01-1.29 | 0.033 |
|  |  | 3 | 7,698 | 688 | 62,192 | 11.1 (10.2-11.9) | 1.38 | 1.21-1.57 | <0.001 |
|  |  | ≥4 | 1,849 | 174 | 14,760 | 11.8 (10.0-13.5) | 1.57 | 1.30-1.89 | <0.001 |
| Patients with diabetes without cardio-renal disease | | 0 | 3,421 | 100 | 29,412 | 3.4 (2.7-4.1) |  |  |  |
|  |  | 1 | 23,948 | 725 | 213,882 | 3.4 (3.1-3.6) | 1.13 | 0.92-1.40 | 0.246 |
|  |  | 2 | 20,201 | 836 | 178,759 | 4.7 (4.4-5.0) | 1.46 | 1.18-1.79 | <0.001 |
|  |  | 3 | 9,566 | 467 | 83,785 | 5.6 (5.1-6.1) | 1.76 | 1.41-2.18 | <0.001 |
|  |  | ≥4 | 2,915 | 201 | 24,962 | 8.1 (6.9-9.2) | 2.79 | 2.19-3.56 | <0.001 |

Hazard ratios were adjusted for age, gender, follow-up, history of cardiovascular disease, and prescriptions for hypoglycemic, antihypertensive, and lipid-lowering therapy.

HR, hazard ratio; CI, confidence interval.
