# Supplementary material for: Risk factor control and cardiovascular events in patients with type 2 diabetes mellitus
Source: PLoS One. 2024 Feb 29;19(2):e0299035. doi: 10.1371/journal.pone.0299035 (PMC10903792; doi:10.1371/journal.pone.0299035)
Supplement: S4 Table — Hazard ratios were adjusted for age, gender, follow-up, history of cardiovascular disease, and prescriptions for hypoglycemic, antihypertensive, and lipid-lowering therapy. HR, hazard ratio; CI, confidence interval. (DOCX) [file pone.0299035.s005.docx]

**S4 Table. The relative risk of heart failure hospitalization in participants according to the degree of risk factor.**

Hazard ratios were adjusted for age, gender, follow-up, history of cardiovascular disease, and prescriptions for hypoglycemic, antihypertensive, and lipid-lowering therapy.

HR, hazard ratio; CI, confidence interval.

|  |  | Uncontrolled risk factors, N | Total  cases | Events | Person-years | Incidence rate per 1000 person-years (95% CI) | HR | 95% CI | P-value |
| --- | --- | --- | --- | --- | --- | --- | --- | --- | --- |
| Total  participants | Subjects without diabetes |  | 290,339 | 1,189 | 2,696,153 | 0.4 (0.4-0.5) |  |  |  |
|  | Patients with diabetes | 0 | 8,280 | 145 | 68,007 | 2.1 (1.8-2.5) | 1.54 | 1.28-1.84 | <0.001 |
|  |  | 1 | 45,253 | 508 | 396,254 | 1.3 (1.2-1.4) | 1.20 | 1.07-1.34 | 0.002 |
|  |  | 2 | 38,348 | 458 | 335,216 | 1.4 (1.2-1.5) | 1.21 | 1.07-1.36 | 0.002 |
|  |  | 3 | 17,264 | 226 | 149,703 | 1.5 (1.3-1.7) | 1.49 | 1.28-1.74 | <0.001 |
|  |  | ≥4 | 4,764 | 64 | 41,076 | 1.6 (1.2-1.9) | 2.09 | 1.61-2.72 | <0.001 |
| Patients with diabetes | | 0 | 8,280 | 145 | 68,007 | 2.1 (1.8-2.5) |  |  |  |
|  |  | 1 | 45,253 | 508 | 396,254 | 1.3 (1.2-1.4) | 0.78 | 0.65-0.94 | 0.009 |
|  |  | 2 | 38,348 | 458 | 335,216 | 1.4 (1.2-1.5) | 0.79 | 0.65-0.95 | 0.013 |
|  |  | 3 | 17,264 | 226 | 149,703 | 1.5 (1.3-1.7) | 0.97 | 0.79-1.20 | 0.775 |
|  |  | ≥4 | 4,764 | 64 | 41,076 | 1.6 (1.2-1.9) | 1.34 | 0.99-1.80 | 0.056 |
| Patients with diabetes with cardio-renal disease | | 0 | 4,859 | 118 | 38,307 | 3.1 (2.5-3.6) |  |  |  |
|  |  | 1 | 21,305 | 422 | 179,833 | 2.3 (2.1-2.6) | 0.86 | 0.70-1.06 | 0.149 |
|  |  | 2 | 18,147 | 347 | 153,643 | 2.3 (2.0-2.5) | 0.79 | 0.64-0.98 | 0.030 |
|  |  | 3 | 7,698 | 180 | 64,216 | 2.8 (2.4-3.2) | 1.05 | 0.83-1.33 | 0.687 |
|  |  | ≥4 | 1,849 | 42 | 15,336 | 2.7 (1.9-3.6) | 1.25 | 0.88-1.79 | 0.217 |
| Patients with diabetes without cardio-renal disease | | 0 | 3,421 | 27 | 29,700 | 0.9 (0.6-1.3) |  |  |  |
|  |  | 1 | 23,948 | 86 | 216,420 | 0.4 (0.3-0.5) | 0.50 | 0.32-0.78 | 0.002 |
|  |  | 2 | 20,201 | 111 | 181,573 | 0.6 (0.5-0.7) | 0.72 | 0.47-1.09 | 0.119 |
|  |  | 3 | 9,566 | 46 | 85,487 | 0.5 (0.4-0.7) | 0.66 | 0.41-1.06 | 0.088 |
|  |  | ≥4 | 2,915 | 22 | 25,740 | 0.9 (0.5-1.2) | 1.34 | 0.76-2.36 | 0.320 |
