# Supplementary material for: Risk factor control and cardiovascular events in patients with type 2 diabetes mellitus
Source: PLoS One. 2024 Feb 29;19(2):e0299035. doi: 10.1371/journal.pone.0299035 (PMC10903792; doi:10.1371/journal.pone.0299035)
Supplement: S5 Table — Hazard ratios were adjusted for age, gender, follow-up, history of cardiovascular disease, and prescriptions for hypoglycemic, antihypertensive, and lipid-lowering therapy. HR, hazard ratio; CI, confidence interval. (DOCX) [file pone.0299035.s006.docx]

**S5 Table. The relative risk of coronary heart disease mortality in participants according to the degree of risk factor.**

|  |  | Uncontrolled risk factors, N | Total  cases | Events | Person-years | Incidence rate per 1000 person-years (95% CI) | HR | 95% CI | P-value |
| --- | --- | --- | --- | --- | --- | --- | --- | --- | --- |
| Total  participants | Subjects without diabetes |  | 290,339 | 795 | 2,699,573 | 0.3 (0.3-0.3) |  |  |  |
|  | Patients with diabetes | 0 | 8,280 | 66 | 68,385 | 1.0 (0.7-1.2) | 1.05 | 0.81-1.36 | 0.725 |
|  |  | 1 | 45,253 | 250 | 397,662 | 0.6 (0.6-0.7) | 0.99 | 0.85-1.16 | 0.924 |
|  |  | 2 | 38,348 | 259 | 336,502 | 0.8 (0.7-0.9) | 1.14 | 0.98-1.33 | 0.095 |
|  |  | 3 | 17,264 | 161 | 150,339 | 1.1 (0.9-1.2) | 1.68 | 1.39-2.02 | <0.001 |
|  |  | ≥4 | 4,764 | 36 | 41,258 | 0.9 (0.6-1.2) | 1.63 | 1.15-2.32 | 0.006 |
| Patients with diabetes | | 0 | 8,280 | 66 | 68,385 | 1.0 (0.7-1.2) |  |  |  |
|  |  | 1 | 45,253 | 250 | 397,662 | 0.6 (0.6-0.7) | 0.92 | 0.70-1.21 | 0.565 |
|  |  | 2 | 38,348 | 259 | 336,502 | 0.8 (0.7-0.9) | 1.06 | 0.81-1.39 | 0.671 |
|  |  | 3 | 17,264 | 161 | 150,339 | 1.1 (0.9-1.2) | 1.57 | 1.17-2.09 | 0.002 |
|  |  | ≥4 | 4,764 | 36 | 41,258 | 0.9 (0.6-1.2) | 1.53 | 1.02-2.31 | 0.042 |
| Patients with diabetes with cardio-renal disease | | 0 | 4,859 | 55 | 38,650 | 1.4 (1.0-1.8) |  |  |  |
|  |  | 1 | 21,305 | 178 | 181,006 | 1.0 (0.8-1.1) | 0.89 | 0.66-1.21 | 0.460 |
|  |  | 2 | 18,147 | 184 | 154,638 | 1.2 (1.0-1.4) | 1.03 | 0.76-1.39 | 0.861 |
|  |  | 3 | 7,698 | 101 | 64,729 | 1.6 (1.3-1.9) | 1.40 | 1.00-1.94 | 0.050 |
|  |  | ≥4 | 1,849 | 25 | 15,464 | 1.6 (1.0-2.3) | 1.62 | 1.00-2.61 | 0.050 |
| Patients with diabetes without cardio-renal disease | | 0 | 3,421 | 11 | 29,735 | 0.4 (0.2-0.6) |  |  |  |
|  |  | 1 | 23,948 | 72 | 216,655 | 0.3 (0.3-0.4) | 1.13 | 0.60-2.13 | 0.713 |
|  |  | 2 | 20,201 | 75 | 181,864 | 0.4 (0.3-0.5) | 1.28 | 0.68-2.41 | 0.448 |
|  |  | 3 | 9,566 | 60 | 85,610 | 0.7 (0.5-0.9) | 2.20 | 1.15-4.19 | 0.017 |
|  |  | ≥4 | 2,915 | 11 | 25,795 | 0.4 (0.2-0.7) | 1.56 | 0.67-3.62 | 0.302 |

Hazard ratios were adjusted for age, gender, follow-up, history of cardiovascular disease, and prescriptions for hypoglycemic, antihypertensive, and lipid-lowering therapy.

HR, hazard ratio; CI, confidence interval.
