# Supplementary material for: Risk factor control and cardiovascular events in patients with type 2 diabetes mellitus
Source: PLoS One. 2024 Feb 29;19(2):e0299035. doi: 10.1371/journal.pone.0299035 (PMC10903792; doi:10.1371/journal.pone.0299035)
Supplement: S6 Table — Hazard ratios were adjusted for age, gender, follow-up, history of cardiovascular disease, and prescriptions for hypoglycemic, antihypertensive, and lipid-lowering therapy. HR, hazard ratio; CI, confidence interval. (DOCX) [file pone.0299035.s007.docx]

**S6 Table. The relative risk of stroke mortality in participants according to the degree of risk factor.**

|  |  | Uncontrolled risk factors, N | Total  cases | Events | Person-years | Incidence rate per 1000 person-years (95% CI) | HR | 95% CI | P-value |
| --- | --- | --- | --- | --- | --- | --- | --- | --- | --- |
| Total  participants | Subjects without diabetes |  | 290,339 | 879 | 2,699,573 | 0.3 (0.3-0.3) |  |  |  |
|  | Patients with diabetes | 0 | 8,280 | 61 | 68,385 | 0.9 (0.7-1.1) | 1.04 | 0.79-1.36 | 0.772 |
|  |  | 1 | 45,253 | 275 | 397,662 | 0.7 (0.6-0.8) | 1.04 | 0.90-1.20 | 0.604 |
|  |  | 2 | 38,348 | 263 | 336,502 | 0.8 (0.7-0.9) | 1.11 | 0.96-1.30 | 0.164 |
|  |  | 3 | 17,264 | 125 | 150,339 | 0.8 (0.7-1.0) | 1.31 | 1.06-1.60 | 0.011 |
|  |  | ≥4 | 4,764 | 39 | 41,258 | 0.9 (0.6-1.2) | 1.90 | 1.36-2.66 | <0.001 |
| Patients with diabetes | | 0 | 8,280 | 61 | 68,385 | 0.9 (0.7-1.1) |  |  |  |
|  |  | 1 | 45,253 | 275 | 397,662 | 0.7 (0.6-0.8) | 0.98 | 0.74-1.29 | 0.873 |
|  |  | 2 | 38,348 | 263 | 336,502 | 0.8 (0.7-0.9) | 1.05 | 0.79-1.39 | 0.736 |
|  |  | 3 | 17,264 | 125 | 150,339 | 0.8 (0.7-1.0) | 1.24 | 0.91-1.68 | 0.181 |
|  |  | ≥4 | 4,764 | 39 | 41,258 | 0.9 (0.6-1.2) | 1.80 | 1.20-2.71 | 0.005 |
| Patients with diabetes with cardio-renal disease | | 0 | 4,859 | 41 | 38,650 | 1.1 (0.7-1.4) |  |  |  |
|  |  | 1 | 21,305 | 214 | 181,006 | 1.2 (1.0-1.3) | 1.26 | 0.90-1.77 | 0.179 |
|  |  | 2 | 18,147 | 164 | 154,638 | 1.1 (0.9-1.2) | 1.08 | 0.77-1.53 | 0.647 |
|  |  | 3 | 7,698 | 81 | 64,729 | 1.3 (1.0-1.5) | 1.39 | 0.95-2.02 | 0.091 |
|  |  | ≥4 | 1,849 | 22 | 15,464 | 1.4 (0.8-2.0) | 1.90 | 1.13-3.21 | 0.016 |
| Patients with diabetes without cardio-renal disease | | 0 | 3,421 | 20 | 29,735 | 0.7 (0.4-1.0) |  |  |  |
|  |  | 1 | 23,948 | 61 | 216,655 | 0.3 (0.2-0.4) | 0.50 | 0.30-0.83 | 0.007 |
|  |  | 2 | 2,0201 | 99 | 181,864 | 0.5 (0.4-0.7) | 0.89 | 0.55-1.45 | 0.647 |
|  |  | 3 | 9,566 | 44 | 85,610 | 0.5 (0.4-0.7) | 0.89 | 0.52-1.51 | 0.651 |
|  |  | ≥4 | 2,915 | 17 | 25,795 | 0.7 (0.3-1.0) | 1.41 | 0.73-2.71 | 0.306 |

Hazard ratios were adjusted for age, gender, follow-up, history of cardiovascular disease, and prescriptions for hypoglycemic, antihypertensive, and lipid-lowering therapy.

HR, hazard ratio; CI, confidence interval.
