# Supplementary material for: Risk factor control and cardiovascular events in patients with type 2 diabetes mellitus
Source: PLoS One. 2024 Feb 29;19(2):e0299035. doi: 10.1371/journal.pone.0299035 (PMC10903792; doi:10.1371/journal.pone.0299035)
Supplement: S7 Table — Hazard ratios were adjusted for age, gender, follow-up, history of cardiovascular disease, and prescriptions for hypoglycemic, antihypertensive, and lipid-lowering therapy. HR, hazard ratio; CI, confidence interval. (DOCX) [file pone.0299035.s008.docx]

**S7 Table. The relative risk of heart failure mortality in participants according to the degree of risk factor.**

|  |  | Uncontrolled risk factors, N | Total  cases | Events | Person-years | Incidence rate per 1000 person-years (95% CI) | HR | 95% CI | P-value |
| --- | --- | --- | --- | --- | --- | --- | --- | --- | --- |
| Total  participants | Subjects without diabetes |  | 290,339 | 222 | 2,699,573 | 0.1 (0.1-0.1) |  |  |  |
|  | Patients with diabetes | 0 | 8,280 | 22 | 68,385 | 0.3 (0.2-0.5) | 1.27 | 0.80-2.00 | 0.313 |
|  |  | 1 | 45,253 | 77 | 397,662 | 0.2 (0.2-0.2) | 0.97 | 0.74-1.27 | 0.812 |
|  |  | 2 | 38,348 | 84 | 336,502 | 0.3 (0.2-0.3) | 1.20 | 0.91-1.57 | 0.194 |
|  |  | 3 | 17,264 | 43 | 150,339 | 0.3 (0.2-0.4) | 1.61 | 1.13-2.31 | 0.009 |
|  |  | ≥4 | 4,764 | 5 | 41,258 | 0.1 (0.0-0.2) | 1.04 | 0.42-2.57 | 0.937 |
| Patients with diabetes | | 0 | 8,280 | 22 | 68,385 | 0.3 (0.2-0.5) |  |  |  |
|  |  | 1 | 45,253 | 77 | 397,662 | 0.2 (0.2-0.2) | 0.72 | 0.45-1.16 | 0.177 |
|  |  | 2 | 38,348 | 84 | 336,502 | 0.3 (0.2-0.3) | 0.90 | 0.56-1.44 | 0.653 |
|  |  | 3 | 17,264 | 43 | 150,339 | 0.3 (0.2-0.4) | 1.20 | 0.72-2.02 | 0.488 |
|  |  | ≥4 | 4,764 | 5 | 41258 | 0.1 (0.0-0.2) | 0.76 | 0.29-2.01 | 0.577 |
| Patients with diabetes with cardio-renal disease | | 0 | 4,859 | 16 | 38,650 | 0.4 (0.2-0.6) |  |  |  |
|  |  | 1 | 21,305 | 60 | 181,006 | 0.3 (0.2-0.4) | 0.87 | 0.50-1.53 | 0.634 |
|  |  | 2 | 18,147 | 63 | 154,638 | 0.4 (0.3-0.5) | 1.04 | 0.60-1.81 | 0.893 |
|  |  | 3 | 7,698 | 32 | 64,729 | 0.5 (0.3-0.7) | 1.42 | 0.77-2.60 | 0.261 |
|  |  | ≥4 | 1,849 | 4 | 15,464 | 0.3 (0.0-0.5) | 0.99 | 0.33-2.99 | 0.986 |
| Patients with diabetes without cardio-renal disease | | 0 | 3,421 | 6 | 29,735 | 0.2 (0.0-0.4) |  |  |  |
|  |  | 1 | 23,948 | 17 | 216,655 | 0.1 (0.0-0.1) | 0.40 | 0.16-1.02 | 0.054 |
|  |  | 2 | 20,201 | 21 | 181,864 | 0.1 (0.1-0.2) | 0.57 | 0.23-1.43 | 0.232 |
|  |  | 3 | 9,566 | 11 | 85,610 | 0.1 (0.1-0.2) | 0.72 | 0.27-1.97 | 0.527 |
|  |  | ≥4 | 2,915 | 1 | 25,795 | 0.0 (0.0-0.1) | 0.33 | 0.04-2.79 | 0.311 |

Hazard ratios were adjusted for age, gender, follow-up, history of cardiovascular disease, and prescriptions for hypoglycemic, antihypertensive, and lipid-lowering therapy.

HR, hazard ratio; CI, confidence interval.
