# Supplementary material for: Risk factor control and cardiovascular events in patients with type 2 diabetes mellitus
Source: PLoS One. 2024 Feb 29;19(2):e0299035. doi: 10.1371/journal.pone.0299035 (PMC10903792; doi:10.1371/journal.pone.0299035)
Supplement: Attachment — Submitted filename: Response to Reviewers.docx [file pone.0299035.s009.docx]

PONE-D-23-37030

Risk Factor Control and Cardiovascular Events in Patients with Type 2 Diabetes Mellitus

PLOS ONE

Dear Dr. Lee,

Thank you for submitting your manuscript to PLOS ONE. After careful consideration, we feel that it has merit but does not fully meet PLOS ONE’s publication criteria as it currently stands. Therefore, we invite you to submit a revised version of the manuscript that addresses the points raised during the review process.

==============================
**ACADEMIC EDITOR:**
==============================

Please submit your revised manuscript by Feb 09 2024 11:59PM. If you will need more time than this to complete your revisions, please reply to this message or contact the journal office at [plosone@plos.org](mailto:plosone@plos.org). When you're ready to submit your revision, log on to https://www.editorialmanager.com/pone/ and select the 'Submissions Needing Revision' folder to locate your manuscript file.

Please include the following items when submitting your revised manuscript:

- A rebuttal letter that responds to each point raised by the academic editor and reviewer(s). You should upload this letter as a separate file labeled 'Response to Reviewers'.
- A marked-up copy of your manuscript that highlights changes made to the original version. You should upload this as a separate file labeled 'Revised Manuscript with Track Changes'.
- An unmarked version of your revised paper without tracked changes. You should upload this as a separate file labeled 'Manuscript'.

If you would like to make changes to your financial disclosure, please include your updated statement in your cover letter. Guidelines for resubmitting your figure files are available below the reviewer comments at the end of this letter.

If applicable, we recommend that you deposit your laboratory protocols in protocols.io to enhance the reproducibility of your results. Protocols.io assigns your protocol its own identifier (DOI) so that it can be cited independently in the future. For instructions see: <https://journals.plos.org/plosone/s/submission-guidelines#loc-laboratory-protocols>. Additionally, PLOS ONE offers an option for publishing peer-reviewed Lab Protocol articles, which describe protocols hosted on protocols.io. Read more information on sharing protocols at <https://plos.org/protocols?utm_medium=editorial-email&utm_source=authorletters&utm_campaign=protocols>.

We look forward to receiving your revised manuscript.

Kind regards,

Hidetaka Hamasaki
Academic Editor
PLOS ONE

Journal requirements:

1. When submitting your revision, we need you to address these additional requirements.

Please ensure that your manuscript meets PLOS ONE's style requirements, including those for file naming. The PLOS ONE style templates can be found at 
https://journals.plos.org/plosone/s/file?id=wjVg/PLOSOne_formatting_sample_main_body.pdf and 
<https://journals.plos.org/plosone/s/file?id=ba62/PLOSOne_formatting_sample_title_authors_affiliations.pdf>.

→ Thank you for your kind comments. We revised the manuscript to conform to the style requirements of PLOS ONE.

2. Thank you for stating the following financial disclosure: 
 [This work was supported by a grant (D.K.S., 2021F-9) from the Korean Diabetes Association.].  
Please state what role the funders took in the study.  If the funders had no role, please state: ""The funders had no role in study design, data collection and analysis, decision to publish, or preparation of the manuscript."" 
If this statement is not correct you must amend it as needed. 
Please include this amended Role of Funder statement in your cover letter; we will change the online submission form on your behalf.

→ We included this amended Role of Funder statement in the cover letter.

3. Please include captions for your Supporting Information files at the end of your manuscript, and update any in-text citations to match accordingly. Please see our Supporting Information guidelines for more information: <http://journals.plos.org/plosone/s/supporting-information>.

→ We included captions for your Supporting Information files at the end of your manuscript.

[Note: HTML markup is below. Please do not edit.]

Reviewers' comments:

Reviewer's Responses to Questions

**Comments to the Author**

1. Is the manuscript technically sound, and do the data support the conclusions?

The manuscript must describe a technically sound piece of scientific research with data that supports the conclusions. Experiments must have been conducted rigorously, with appropriate controls, replication, and sample sizes. The conclusions must be drawn appropriately based on the data presented.

Reviewer #1: Yes

Reviewer #2: Partly

2. Has the statistical analysis been performed appropriately and rigorously?

Reviewer #1: Yes

Reviewer #2: Yes

3. Have the authors made all data underlying the findings in their manuscript fully available?

The [PLOS Data policy](http://www.plosone.org/static/policies.action#sharing) requires authors to make all data underlying the findings described in their manuscript fully available without restriction, with rare exception (please refer to the Data Availability Statement in the manuscript PDF file). The data should be provided as part of the manuscript or its supporting information, or deposited to a public repository. For example, in addition to summary statistics, the data points behind means, medians and variance measures should be available. If there are restrictions on publicly sharing data—e.g. participant privacy or use of data from a third party—those must be specified.

Reviewer #1: Yes

Reviewer #2: Yes

4. Is the manuscript presented in an intelligible fashion and written in standard English?

PLOS ONE does not copyedit accepted manuscripts, so the language in submitted articles must be clear, correct, and unambiguous. Any typographical or grammatical errors should be corrected at revision, so please note any specific errors here.

Reviewer #1: Yes

Reviewer #2: No

5. Review Comments to the Author

Please use the space provided to explain your answers to the questions above. You may also include additional comments for the author, including concerns about dual publication, research ethics, or publication ethics. (Please upload your review as an attachment if it exceeds 20,000 characters)

Reviewer #1: The present study was aimed at risk factor control and cardiovascular events in patients with type 2 diabetes mellitus. Controlling cardiovascular risk factors reduced CVD events in diabetic patients, especially in low CVD risk group. There was a strong association between the degree of risk control and risk of CVD mortality in diabetic patients with baseline high CVD risk. These findings highlight that early and more intensive cardiovascular risk factor interventions, which help reduce cardiovascular events and mortality in Korean patients with diabetes. The data are reliable and the article is well written. The study is closely integrated with the clinic and has certain innovation.
It would be more comprehensive to add information about pharmacological treatment with antiplatelet drugs. The study was a retrospective study, and future cohort studies should be conducted to support the findings of this study.

→ Thank you for your kind comments. “We did not consider risk factor changes during the follow-up and treatment with antiplatelet drugs that could influence the prevalence of CVD events.” was inserted in the Discussion.

Reviewer #2: Authors analyzed national insurance data source and data from one hospital to understand CVD events and risk factors in Korean diabetic patients. The study helps to develop treatment options for diabetes patients based on the risk factors. I have several critiques authors have to address.


1. Is there any reason why authors categorize previous CVD history or eGFR <45 as a high CVD risk in the diabetes population? Is this in the guideline?

→ Thank you for your kind comments. We changed “High CVD risk” into “cardio-renal disease”. We inserted “Baseline cardio-renal disease was defined as prior CVD or estimated glomerular filtration rate (eGFR) < 45 mL/min/1.73 m^2^ at cohort entry.” in the Materials and methods.

2. If the above answer is no, the term of CVD risk is confusing. Authors often use CVD risk, risk factors and CVD events in a sentence. It is really difficult to comprehend authors intentions.

→ Thank you for your kind comments. We deleted the term high or low CVD risk. Instead, we stratified the diabetic patients according to the presence of cardio-renal disease.

3. Is CDM analysis necessary? Authors admit the limitation of the data such as smaller sample size, tracking mortality and so on. Authors spend significant amounts of CDM analysis in the manuscript. This makes the discussion complicated and wordy. It seems NHIS data is sufficient to lead authors conclusions.

→ Thank you for your kind comments. We deleted the data of CDM.

4. Results section is confusing. Readers have to go back and forth among tables. Please rewrite the section or tables to make them in order.

→ Thank you for your kind comments. We deleted the data of CDM and rewrited the section to make them in order.

5. Please use standard English. There are many places where I do not comprehend easily what authors try to convey. I would recommend English proofing service to polish manuscript. Below are the examples.

→ Thank you for your kind comments. We received full English editing.

• Authors often use “without poorly controlled risk factors”. Do those simply go with “without risk factors” or “with controlled risk factors”?

→Thank you for your kind comments. We changed “without poorly controlled risk factors” into “without risk factors”.

• Line 245-246, I do not understand the authors intention to use the sentence here.

→We removed “Diabetic patients with severe disabilities or who were hospitalized might be excluded from the NHIS database.” because CDM data was excluded from the paper.

• Line 249-252, I do not understand the sentence.

→ We removed “It was possible that the risk of CVD did not decrease in patients without diabetes because of other comorbidities or advanced diseases compared to those with diabetes without poorly controlled risk factors per the CDM.” because CDM data was excluded from the paper.

• Line 291-294, I do not understand the sentence.

→We removed the sentence.

• Line 316-317, the sentence is difficult to comprehend. Is this “diabetic patients who do not have any risk factors are only 7% in the population”?

→ We changed the sentence to “Patients with diabetes without any risk factors constituted only 7% of the population.”

• Line 320-322, I do not understand the authors intention for the sentence.

→ We changed “Risk factor control must be promoted to aid in the real-world management of people with diabetes.” to “Therefore, risk factors must be well managed in patients with diabetes.”

• Line 323-326, the sentence is too long and confusing. Several “and” and “or” are used in one sentence.

→ We changed the sentence to “This is the first study to evaluate the association between cardiovascular risk factor control and the incidence of CVD events or mortality according to the prevalence of cardio-renal disease in Korean patients with diabetes.”

• Line 355-357, repetitive.

→ We removed the sentence “It was difficult to determine the actual duration of diabetes with NHIS or CDM data because the subjects included in the study might not have been diagnosed with diabetes at the beginning of the study.”

6. PLOS authors have the option to publish the peer review history of their article ([what does this mean?](https://journals.plos.org/plosone/s/editorial-and-peer-review-process#loc-peer-review-history)). If published, this will include your full peer review and any attached files.
If you choose “no”, your identity will remain anonymous but your review may still be made public.

**Do you want your identity to be public for this peer review?** For information about this choice, including consent withdrawal, please see our [Privacy Policy](https://www.plos.org/privacy-policy).

Reviewer #1: No

Reviewer #2: No

[NOTE: If reviewer comments were submitted as an attachment file, they will be attached to this email and accessible via the submission site. Please log into your account, locate the manuscript record, and check for the action link "View Attachments". If this link does not appear, there are no attachment files.]

While revising your submission, please upload your figure files to the Preflight Analysis and Conversion Engine (PACE) digital diagnostic tool, <https://pacev2.apexcovantage.com/>. PACE helps ensure that figures meet PLOS requirements. To use PACE, you must first register as a user. Registration is free. Then, login and navigate to the UPLOAD tab, where you will find detailed instructions on how to use the tool. If you encounter any issues or have any questions when using PACE, please email PLOS at [figures@plos.org](mailto:figures@plos.org). Please note that Supporting Information files do not need this step.

→ We confirm that the figure meets PLOS requirements although the figure was supporting information.
